# Distinguishing Characteristics between Pandemic 2009–2010 Influenza A (H1N1) and Other Viruses in Patients Hospitalized with Respiratory Illness

**DOI:** 10.1371/journal.pone.0024734

**Published:** 2011-09-16

**Authors:** Philip A. Chan, Leonard A. Mermel, Sarah B. Andrea, Russell McCulloh, John P. Mills, Ignacio Echenique, Emily Leveen, Natasha Rybak, Cheston Cunha, Jason T. Machan, Terrance T. Healey, Kimberle C. Chapin

**Affiliations:** 1 Division of Infectious Diseases, Rhode Island Hospital, Providence, Rhode Island, United States of America; 2 Department of Medicine, Alpert Medical School of Brown University, Providence, Rhode Island, United States of America; 3 Department of Pathology, Rhode Island Hospital, Providence, Rhode Island, United States of America; 4 Division of Pediatric Infectious Diseases, Rhode Island Hospital, Providence, Rhode Island, United States of America; 5 Department of Pediatrics, Alpert Medical School of Brown University, Providence, Rhode Island, United States of America; 6 Department of Orthopedics and Surgery, Rhode Island Hospital, Providence, Rhode Island, United States of America; 7 Department of Radiology, Rhode Island Hospital, Providence, Rhode Island, United States of America; Johns Hopkins University - Bloomberg School of Public Health, United States of America

## Abstract

**Background:**

Differences in clinical presentation and outcomes among patients infected with pandemic 2009 influenza A H1N1 (pH1N1) compared to other respiratory viruses have not been fully elucidated.

**Methodology/Principal Findings:**

A retrospective study was performed of all hospitalized patients at the peak of the pH1N1 season in whom a single respiratory virus was detected by a molecular assay targeting 18 viruses/subtypes (RVP, Luminex xTAG). Fifty-two percent (615/1192) of patients from October, 2009 to December, 2009 had a single respiratory virus (291 pH1N1; 207 rhinovirus; 45 RSV A/B; 37 parainfluenza; 27 adenovirus; 6 coronavirus; and 2 metapneumovirus). No seasonal influenza A or B was detected. Individuals with pH1N1, compared to other viruses, were more likely to present with fever (92% & 70%), cough (92% & 86%), sore throat (32% & 16%), nausea (31% & 8%), vomiting (39% & 30%), abdominal pain (14% & 7%), and a lower white blood count (8,500/L & 13,600/L, all p-values<0.05). In patients with cough and gastrointestinal complaints, the presence of subjective fever/chills independently raised the likelihood of pH1N1 (OR 10). Fifty-five percent (336/615) of our cohort received antibacterial agents, 63% (385/615) received oseltamivir, and 41% (252/615) received steroids. The mortality rate of our cohort was 1% (7/615) and was higher in individuals with pH1N1 compared to other viruses (2.1% & 0.3%, respectively; p = 0.04).

**Conclusions/Significance:**

During the peak pandemic 2009–2010 influenza season in Rhode Island, nearly half of patients admitted with influenza-like symptoms had respiratory viruses other than influenza A. A high proportion of patients were treated with antibiotics and pH1N1 infection had higher mortality compared to other respiratory viruses.

## Introduction

Viral respiratory illnesses are responsible for large numbers of hospital admissions each year leading to substantial morbidity and mortality [1]. The etiologic agents include a diverse group of viruses, such as influenza A which is responsible for intermittent pandemics [2]. Reassortment of swine-origin and human strains led to circulating pH1N1 [3], [4] and a significant increase in hospital admissions during the 2009–2010 influenza season.

Timely identification of influenza is important as the administration of neuraminidase inhibitors may limit duration and severity of illness if given early [5]. Rapid tests were found to be insensitive in the diagnosis of pH1N1 [6] and unable to subtype the influenza virus. Molecular techniques replaced some of these tests, but the availability, expense and technical training limited widespread use of this technology [7]. Therefore, many clinicians relied on clinical symptoms to diagnose influenza during the pandemic [8].

The classic influenza-like illness (ILI), defined as fever and cough and/or sore throat, is often used to distinguish influenza from other respiratory viruses. However, other viruses such as respiratory syncytial virus (RSV A/B), rhinovirus, parainfluenza, adenovirus, metapneumovirus, and coronavirus, can cause a similar illness and circulate at the same time as influenza [1], [9]. Using ILI symptoms to diagnose influenza is neither sensitive nor specific [10], [11]. Other symptoms reported during the pandemic included gastrointestinal complaints [12]–[19], leukopenia [14], [20], [21], elevated aminotransferase levels [14], thrombocytopenia and other laboratory abnormalities [14], [20]–[24]. Although the clinical characteristics of pH1N1 infection may be similar to seasonal influenza [25], [26], there is scant data in the literature comparing pH1N1 with other respiratory viruses.

The inability to reliably diagnose a viral respiratory infection such as influenza A, often leads to coverage of possible bacterial etiologies [27]. Overuse of antibiotics is not without consequence and can lead to complications including *Clostridium difficile* infection and high rates of resistance [28]. Thus, an accurate diagnosis of influenza and other respiratory viral infections is important to avoid overuse of antibacterial agents and direct appropriate antiviral therapy.

In response to the diagnostic challenges presented by influenza infection, our hospital system instituted a polymerase chain reaction (PCR)-based molecular panel that was able to identify 18 different respiratory viruses. The aim of this study was to examine differences in clinical, laboratory and radiographic findings between pH1N1 and other respiratory viruses with the goal to assist clinicians in more effectively diagnosing and treating pH1N1. To our knowledge, this is the first study to directly compare clinical parameters of pH1N1 to other respiratory viruses using a sensitive molecular diagnostic methodology in a large cohort.

## Results

**Figure 1:**
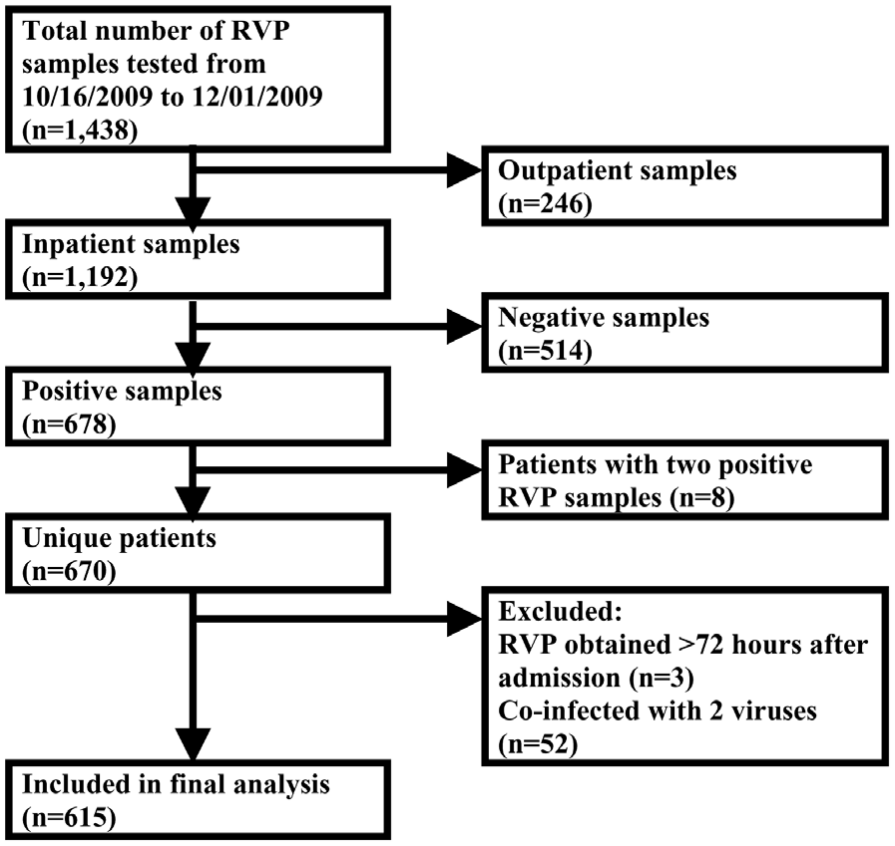
Study inclusion algorithm.

During our peak pH1N1 season, 1,438 RVP samples were collected. Of these, 1192 were from inpatients (340 samples in patients <5 years, 240 samples 5–18 years, and 612 samples >19 years). Six-hundred and fifteen patients with positive results were included in the final analysis (Figure 1) with a mean age of 20 years (range: 0–97 years). Forty-seven percent of patients had pH1N1 and 53% had another respiratory virus with rhinovirus being the second most prevalent in the population analyzed (34%, Table 1). Fewer patients with pH1N1 were under the age of five years compared to those with other viruses and individuals with pH1N1 were less likely to have cardiac co-morbidities, malignancy, or be admitted from a nursing home. Individuals with pH1N1 were more likely to report a sick contact or to use tobacco.

**Table 1:** Characteristics and demographics of patients presenting with pandemic influenza A (pH1N1) compared to other respiratory viruses.

	Total(n = 615)	Influenza A, H1N1(n = 291)	All Others(n = 324)	Rhinovirus(n = 207)	RSV(n = 45)	Adenovirus(n = 27)	Parainfluenza(n = 37)	Coronavirus(n = 6)	Metapneumovirus(n = 2)	p-value	OR^a^
**Age (years)**											
**5<**	37.9% (233)	21.3% (62)	52.8% (171)	47.8% (99)	84.4% (38)	44.4% (12)	48.6% (18)	50.0% (3)	50.0% (1)	<0.01	-
**5 to 18**	23.6% (145)	32.3% (94)	15.7% (51)	16.4% (34)	6.7% (3)	22.2% (6)	21.6% (8)	0% (0)	0% (0)		1.01 [0.52–1.95]
**19 to 59**	29.4% (181)	41.2% (120)	18.8% (61)	22.2% (46)	2.2% (1)	29.6% (8)	10.8% (4)	16.7% (1)	50.0% (1)		5.43 [3.55–8.29]
**≥60**	9.1% (56)	5.2% (15)	12.7% (41)	13.5% (28)	6.7% (3)	3.7% (1)	18.9% (7)	33.3% (2)	0% (0)		5.08 [3.25–7.96]
**Gender**											
**Male**	53.5% (329)	52.9% (154)	54.0% (175)	52.2% (108)	68.9% (31)	55.6% (15)	45.9% (17)	66.7% (4)	0% (0)	0.79	0.96 [0.70–1.32]
**Female**	46.5% (286)	47.1% (137)	46.0% (149)	47.8% (99)	31.1% (14)	44.4% (12)	54.1% (20)	33.3% (2)	100.0% (2)		
**Co-morbidities**											
**Respiratory** b	44.7% (275)	46.7% (136)	42.9% (139)	48.3% (100)	35.6% (16)	37.0% (10)	32.4% (12)	0% (0)	50.0% (1)	0.34	1.17 [0.85–1.61]
**Asthma/COPD**	37.4% (230)	39.5% (115)	35.5% (115)	42.0% (87)	22.2% (10)	37.0% (10)	21.6% (8)	0% (0)	0% (0)	0.30	1.19 [0.86–1.65]
**Hepatic** c	2.9% (18)	4.1% (12)	1.9% (6)	2.9% (6)	0% (0)	0% (0)	0% (0)	0% (0)	0% (0)	0.10	2.28 [0.84–6.15]
**Renal** d	2.4% (15)	2.4% (7)	2.5% (8)	2.4% (5)	2.2% (1)	3.7% (1)	2.7% (1)	0% (0)	0% (0)	0.96	0.97 [0.35–2.72]
**Malignancy**	6.0% (37)	2.7% (8)	9.0% (29)	9.2% (19)	4.4% (2)	7.4% (2)	10.8% (4)	33.3% (2)	0% (0)	<0.01	0.29 [0.13–0.64]
**Neurological** e	11.2% (69)	12.7% (37)	9.9% (32)	8.7% (18)	11.1% (5)	0% (0)	18.9% (7)	33.3% (2)	0% (0)	0.27	1.33 [0.81–2.20]
**Cardiac**	12.5% (77)	9.6% (28)	15.1% (49)	16.4% (34)	8.9% (4)	7.4% (2)	18.9% (7)	16.7% (1)	50.0% (1)	0.04	0.60 [0.37–0.98]
**HIV** f	1.8% (11)	1.4% (4)	2.2% (7)	3.4% (7)	0% (0)	0% (0)	0% (0)	0% (0)	0% (0)	0.46	0.63 [0.18–2.18]
**Immuno-compromised** g	6.2% (38)	4.8% (14)	7.4% (24)	9.2% (19)	2.2% (1)	0% (0)	5.4% (2)	16.7% (1)	50.0% (1)	0.18	0.63 [0.32–1.25]
**Other**											
**Tobacco**	15.6% (96)	21.0% (61)	10.8% (35)	11.1% (23)	2.2% (1)	25.9% (7)	10.8% (4)	0% (0)	0% (0)	<0.01	2.19 [1.40–3.44]
**Nursing Home**	2.1% (13)	0.7% (2)	3.4% (11)	5.3% (11)	0% (0)	0% (0)	0% (0)	0% (0)	0% (0)	0.02	0.20 [0.04–0.90]
**Sick Contacts**	34.0% (209)	43.0% (125)	25.9% (84)	24.2% (50)	28.9% (13)	25.9% (7)	29.7% (11)	33.3% (2)	50.0% (1)	<0.01	2.15 [1.53–3.02]
**Pregnant**	1.3% (8)	1.4% (4)	1.2% (4)	1.9% (4)	0% (0)	0% (0)	0% (0)	0% (0)	0% (0)	0.27	1.12 [0.28–4.50]

a Odds ratios for age categories are compared to individuals <5 years-old, otherwise odds ratios are comparing individuals with pandemic influenza A (H1N1) to those with another respiratory virus;

b Congenital malformations, asthma, chronic obstructive pulmonary disease (COPD), chronic lung disease, previous serious or multiple lung infection/disease/intubation, cystic fibrosis, history of reactive airway disease or wheezing;

c Cirrhosis, end-stage liver disease, hepatitis, congenital abnormalities;

d Chronic kidney disease, end-stage renal disease, congenital disease, nephrotic syndrome, transplant recipient; **Malignancy** = leukemia, bladder, breast, head and neck, lung, lymphoma, medulloblastoma, prostate, multiple myeloma, neuroblastoma, ovarian, sarcoma;

e Seizure disorder, dementia, developmental delay, cerebral palsy, stroke, congenital, neuromuscular, trauma/surgery; **Cardiac disease** = arrhythmia, coronary artery disease, heart failure, congenital, valvular disease, cardiomyopathy, myocardial infarction;

f Human Immunodeficiency Virus;

g Systemic steroid use, transplant recipient, chemotherapy, human immunodeficiency virus (HIV).

Individuals with pH1N1 were more likely to present with the following symptoms when compared to those with other respiratory viruses: subjective fever or chills, sore throat, nausea, vomiting, abdominal pain, weakness, fatigue, headache, myalgias, and chest pain. Patients with other respiratory viruses were more likely to present with changes in mental status including dizziness or lethargy (Table 2).

**Table 2:** Signs and symptoms of patients with pandemic 2009 influenza A (pH1N1) compared to other respiratory viruses.

	Total(n = 615)	Influenza A, H1N1(n = 291)	All Others(n = 324)	Rhinovirus(n = 207)	RSV(n = 45)	Adenovirus(n = 27)	Parainfluenza(n = 37)	Coronavirus(n = 6)	Metapneumovirus(n = 2)	p-value	OR
**Fever**	80.2% (493)	91.8% (267)	69.8% (226)	63.8% (132)	77.8% (35)	92.6% (25)	75.7% (28)	66.7% (4)	100.0% (2)	<0.01	4.82 [2.98–7.80]
**Mental Status**	30.6% (188)	24.7% (72)	35.8% (116)	30.9% (64)	48.9% (22)	40.7% (11)	45.9% (17)	33.3% (2)	0% (0)	<0.01	0.59 [0.42–0.84]
**Weakness**	17.2% (106)	26.8% (78)	8.6% (28)	7.2% (15)	6.7% (3)	11.1% (3)	13.5% (5)	33.3% (2)	0% (0)	<0.01	3.87 [2.43–6.17]
**Fatigue**	15.0% (92)	19.6% (57)	10.8% (35)	9.2% (19)	15.6% (7)	14.8% (4)	10.8% (4)	16.7% (1)	0% (0)	<0.01	2.01 [1.28–3.17]
**Otitis Media**	4.4% (27)	4.1% (12)	4.6% (15)	4.8% (10)	2.2% (1)	7.4% (2)	0% (0)	16.7% (1)	50.0% (1)	0.76	0.89 [0.41–1.93]
**Conjunctivitis**	2.0% (12)	1.0% (3)	2.8% (9)	2.4% (5)	2.2% (1)	7.4% (2)	2.7% (1)	0% (0)	0% (0)	0.12	0.37 [0.10–1.36]
**Cough**	88.5% (544)	91.1% (265)	86.1% (279)	85.0% (176)	86.7% (39)	85.2% (23)	94.6% (35)	66.7% (4)	100.0% (2)	0.06	1.64 [0.99–2.74]
**Nasal Symptoms**	56.7% (349)	56.7% (165)	56.8% (184)	52.2% (108)	71.1% (32)	70.4% (19)	54.1% (20)	66.7% (4)	50.0% (1)	0.98	1.00 [0.72–1.37]
**Sore Throat**	23.6% (145)	31.6% (92)	16.4% (53)	17.4% (36)	4.4% (2)	11.1% (3)	24.3% (9)	33.3% (2)	50.0% (1)	<0.01	2.36 [1.61–3.47]
**Headache**	19.7% (121)	29.6% (86)	10.8% (35)	13.0% (27)	0% (0)	7.4% (2)	13.5% (5)	16.7% (1)	0% (0)	<0.01	3.46 [2.25–5.34]
**Myalgias**	20.7% (127)	31.6% (92)	10.8% (35)	11.1% (23)	2.2% (1)	25.9% (7)	10.8% (4)	0% (0)	0% (0)	<0.01	3.82 [2.49–5.86]
**Arthralgias**	2.0% (12)	3.1% (9)	0.9% (3)	1.0% (2)	0% (0)	0% (0)	2.7% (1)	0% (0)	0% (0)	0.05	3.42 [0.92–12.74]
**Chest Pain**	16.4% (101)	23.4% (68)	10.2% (33)	11.6% (24)	4.4% (2)	7.4% (2)	10.8% (4)	0% (0)	50.0% (1)	<0.01	2.69 [1.71–4.22]
**Dyspnea**	58.7% (361)	52.9% (154)	63.9% (207)	64.7% (134)	73.3% (33)	59.3% (16)	59.5% (22)	33.3% (2)	0% (0)	<0.01	0.64 [0.46–0.88]
**Wheezing**	28.0% (172)	25.8% (75)	29.9% (97)	32.9% (68)	31.1% (14)	29.6% (8)	16.2% (6)	16.7% (1)	0% (0)	0.25	0.81 [0.57–1.16]
**Nausea**	19.0% (117)	31.3% (91)	8.0% (26)	10.1% (21)	4.4% (2)	7.4% (2)	2.7% (1)	0% (0)	0% (0)	<0.01	5.22 [3.26–8.35]
**Vomiting**	34.5% (212)	39.2% (114)	30.2% (98)	26.1% (54)	40.0% (18)	40.7% (11)	37.8% (14)	16.7% (1)	0% (0)	0.02	1.49 [1.06–2.07]
**Abdominal Pain**	10.7% (66)	14.4% (42)	7.4% (24)	7.7% (16)	0% (0)	14.8% (4)	5.4% (2)	33.3% (2)	0% (0)	<0.01	2.11 [1.24–3.58]
**Diarrhea**	13.0% (80)	15.8% (46)	10.5% (34)	7.2% (15)	11.1% (5)	22.2% (6)	21.6% (8)	0% (0)	0% (0)	0.05	1.60 [1.00–2.58]
**Anorexia**	37.6% (231)	38.5% (112)	36.7% (119)	32.4% (67)	46.7% (21)	37.0% (10)	48.6% (18)	33.3% (2)	50.0% (1)	0.65	1.08 [0.78–1.50]

OR = odds ratio.

On presentation to the emergency room, patients with pH1N1 exhibited a higher maximum temperature, lower maximum heart rate, respiratory rate, systolic blood pressure, and oxygen saturation (Table 3). Patients with pH1N1 were more likely to have lower white blood counts, platelet counts, and potassium levels. Alternatively, patients with pH1N1 were more likely to have higher hemoglobin/hematocrit and albumin levels.

**Table 3:** Clinical characteristics of patients with pandemic influenza A (pH1N1) compared to other respiratory viruses.

	Pandemic Influenza A (H1N1)			Other Respiratory Viruses			
	Mean [95% CI]	Min	Max	Mean [95% CI]	Min	Max	p-value
**Duration of Symptoms (days)**	3.3 [2.8–3.9]	1.0	60.0	4.0 [3.4–4.5]	1.0	56.0	0.10
**Max Temperature (^o^F)**	100.9 [100.7–101.1]	96.0	106.5	100.2 [99.9–100.4]	92.4	110.3	<0.01
**Max Heart Rate (/min)**	128.6 [125.1–132.0]	65.0	220.0	140.8 [136.8–144.7]	19.0	226	<0.01
**Max Respiratory Rate (/min)**	31.5 [29.9–33.1]	2.0	80.0	39.0 [37.0–41.1]	14.0	168.0	<0.01
**Lowest SBP** a **(mmHG)**	105.7 [103.5–108.0]	43.0	176.0	111.1 [108.5–113.6]	11.0	202.0	<0.01
**Corresponding DBP** b **(mmHG)**	60.1 [58.6–61.7]	12.0	98.0	64.9 [63.1–66.6]	0.0	133.0	<0.01
**Lowest Oxygen Saturation (%)**	93.9 [93.2–94.7]	30.0	100.0	95.2 [94.8–95.6]	73.0	100.0	<0.01
**Sodium (meq/L)**	136.8 [136.4–137.2]	127.0	146.0	136.7 [136.1–137.4]	114.0	144.0	0.92
**Potassium (meq/L)**	3.9 [3.8–4.0]	2.6	6.1	4.1 [4.0–4.1]	2.5	6.0	<0.01
**Bicarbonate (meq/L)**	24.3 [23.9–24.8]	13.0	36.0	25.6 [24.0–27.2]	12.0	107.0	0.13
**Chloride (meq/L)**	102.6 [102.0–103.1]	89.0	114.0	101.8 [100.2–103.4]	21.0	116.0	0.36
**Creatinine (mg/dl)**	0.94 [0.77–1.11]	0.13	10.25	0.95 [0.58–1.32]	0.09	28.0	0.95
**Blood urea nitrogen (mg/dl)**	13.2 [11.4–14.9]	2.0	118.0	12.6 [11.3–13.8]	0.53	60.0	0.59
**Glucose (mg/dl)**	135.6 [126.9–144.4]	51.0	575.0	131.0 [123.1–138.9]	0.66	377.0	0.44
**White blood count (×10^3^/ml)**	8.5 [8.1–9.0]	0.7	21.5	13.6 [12.3–14.9]	0.20	86.5	<0.01
**Bands (%)**	2.8 [2.0–3.6]	0.0	45.0	2.5 [1.8–3.2]	0.0	36.0	0.61
**Hemoglobin (g/dl)**	13.0 [12.8–13.3]	7.2	18.3	12.2 [11.9–12.5]	6.3	17.8	<0.01
**Hematocrit (%)**	38.0 [37.3–38.7]	21.8	52.1	35.9 [35.1–36.8]	19.1	51.7	<0.01
**Platelets (×10^3^/ml)**	229.7 [218.6–240.8]	13.0	539.0	288.7 [271.2–306.2]	16.0	700.0	<0.01
**Lactate (meq/L)**	2.4 [1.3–3.5]	0.4	23.6	2.0 [1.5–2.4]	0.6	7.4	0.44
**AST (IU/L)**	52.0 [39.5–64.4]	14.0	332.0	133.3 [−0.12–266.8]	11.0	3273.0	0.23
**ALT (IU/L)**	36.6 [29.3–44.0]	9.0	148.0	122.2 [10.7–233.7]	8.0	2560.0	0.13
**Total Bilirubin (mg/dl)**	0.8 [0.7–0.9]	0.2	2.0	1.2 [0.7–1.6]	0.2	11.2	0.08
**Direct Bilirubin (mg/dl)**	0.2 [0.1–0.2]	0.1	0.8	0.2 [0.1–0.3]	0.1	1.4	0.21
**Alkaline Phosphate (IU/L)**	95.2 [79.1–111.2]	31.0	277.0	114.2 [91.0–137.4]	48.0	477.0	0.18
**Protein (g/dl)**	6.7 [6.5–6.9]	5.0	9.0	6.5 [6.2–6.7]	4.1	8.8	0.13
**Albumin (g/dl)**	3.5 [3.3–3.6]	2.2	4.9	3.2 [2.9–3.4]	1.5	4.3	0.02
**PTT (sec)**	32.9 [27.4–38.5]	22.3	75.5	29.0 [23.5–34.4]	11.8	41.9	0.31
**PT (sec)**	15.8 [13.2–18.4]	11.5	34.9	18.8 [13.3–24.3]	11.5	49.6	0.31
**INR**	1.4 [1.1–1.7]	0.9	3.6	1.5 [1.1–1.8]	0.9	3.1	0.80
**Creatine Kinase (IU//L)**	304.3 [166.4–442.1]	15.0	2548.0	304.6 [37.9–571.2]	14.0	2368.0	1.00
**MB Fraction (%)**	1.7 [0.5–2.9]	0.0	15.2	3.6 [−0.04–7.2]	0.0	18.7	0.30
**Troponin I (ng/ml)**	0.24 [0.10–0.37]	0.08	2.8	0.82 [−0.15–1.78]	0.03	9.0	0.23

a Systolic blood pressure;

b Diastolic blood pressure; CI = confidence intervals; PT = prothrombin time; PTT = partial thromboplastin time; INR = international normalized ratio; AST = aspartate aminotransferase; ALT = alanine aminotransferase.

Of the 529 patients who received a chest radiograph, a greater number of patients with pH1N1 had no acute findings compared to other respiratory viruses (Table 4). Other respiratory viruses were more likely to have an interstitial opacity consistent with viral infection on chest radiograph. Thirty percent (161/529) of patients with a chest radiograph had focal or multi-focal airspace findings.

**Table 4:** Chest radiograph characteristics of patients with pandemic influenza A (pH1N1) compared to other respiratory viruses.

	Total(n = 529)	Influenza A, H1N1(n = 257)	All Others(n = 272)	Rhinovirus(n = 177)	RSV(n = 35)	Adenovirus(n = 26)	Parainfluenza(n = 27)	Coronavirus(n = 5)	Metapneumovirus(n = 2)	p-value	OR
**NAD** a	51.6% (273)	59.1% (152)	44.5% (121)	45.8% (81)	31.4% (11)	38.5% (10)	51.9% (14)	100.0% (5)	0% (0)	<0.01	1.81 [1.28–2.55]
**IO** b	15.9% (84)	10.9% (28)	20.6% (56)	21.5% (38)	37.1% (13)	7.7% (2)	11.1% (3)	0% (0)	0% (0)	<0.01	0.47 [0.29–0.77]
**FASD** c	18.7% (99)	18.7% (48)	18.8% (51)	16.4% (29)	20.0% (7)	30.8% (8)	22.2% (6)	0% (0)	50.0% (1)	0.98	1.00 [0.64–1.54]
**MFASD** d	9.6% (51)	7.4% (19)	11.8% (32)	10.7% (19)	11.4% (4)	23.1% (6)	7.4% (2)	0% (0)	50.0% (1)	0.09	0.60 [0.33–1.09]
**Effusion**	2.1% (11)	1.9% (5)	2.2% (6)	2.3% (4)	2.9% (1)	0% (0)	3.7% (1)	0% (0)	0% (0)	0.83	0.88 [0.27–2.92]

a No acute disease;

b Interstitial Opacity consistent with viral disease;

c focal air-space disease;

d multi-focal air-space disease.

Other categories included edema (N = 16), collapse (N = 1), pneumomedistinum (N = 3). Patients could have multiple findings on chest radiograph (i.e. MFASD and effusion). OR = odds ratio.

Most patients with pH1N1 (79.0%) received oseltamivir. More than half received antibacterial agents, and one-third received steroids (Table 5). Of the total cohort, only 9.6% had a sputum sample of which 27% were positive for a potential pathogenic microorganism. Forty-four percent (157/357) of patients with no evidence of acute disease or interstitial opacities indicative of viral infection on chest radiograph received antibacterial agents. Forty-six percent of the total cohort had blood sent for culture during their hospitalization, of which 2.5% grew a potential pathogenic microbe (i.e., coagulase-negative staphylococci and other potential skin contaminants were excluded). Twelve percent of the total cohort had a *Legionella* urine antigen test performed and all were negative.

**Table 5:** Treatment and outcomes of patients with pandemic influenza A (pH1N1) compared to other respiratory viruses.

	Total(n = 615)	Influenza A, H1N1(n = 291)	All Others(n = 324)	Rhinovirus(n = 207)	RSV(n = 45)	Adenovirus(n = 27)	Parainfluenza(n = 37)	Coronavirus(n = 6)	Metapneumovirus(n = 2)	p-value	OR
**Oseltamivir**	62.6% (385)	79.0% (230)	47.8% (155)	44.4% (92)	53.3% (24)	55.6% (15)	56.8% (21)	33.3% (2)	50.0% (1)	<0.01	4.11 [2.88–5.87]
**Other antiviral**	0.8% (5)	1.4% (4)	0.3% (1)	0% (0)	2.2% (1)	0% (0)	0% (0)	0% (0)	0% (0)	0.14	4.50 [0.50–40.51]
**Antibacterial agents**	54.6% (336)	55.0% (160)	54.3% (176)	52.7% (109)	46.7% (21)	77.8% (21)	54.1% (20)	50.0% (3)	100.0% (2)	0.87	1.03 [0.75–1.41]
**Steroids**	41.0% (252)	34.4% (100)	46.9% (152)	52.7% (109)	46.7% (21)	37.0% (10)	27.0% (10)	16.7% (1)	50.0% (1)	<0.01	0.59 [0.43–0.82]
**ICU Admission**	16.9% (104)	16.2% (47)	17.6% (57)	18.4% (38)	22.2% (10)	14.8% (4)	10.8% (4)	16.7% (1)	0% (0)	0.56	−
**Intubation**	3.7% (23)	3.4% (10)	4.0% (13)	3.9% (8)	4.4% (2)	7.4% (2)	0% (0)	16.7% (1)	0% (0)	0.71	0.85 [0.37–1.97]
**Bi-pap**	3.6% (22)	5.2% (15)	2.2% (7)	1.4% (3)	2.2% (1)	3.7% (1)	0% (0)	16.7% (1)	50.0% (1)	0.046	2.46 [0.99–6.12
**Nebulizers or Inhalers**	52.5% (323)	46.7% (136)	57.7% (187)	59.9% (124)	75.6% (34)	44.4% (12)	40.5% (15)	16.7% (1)	50.0% (1)	0.02	−
**Pressors**	1.8% (11)	2.7% (8)	0.9% (3)	1.0% (2)	0% (0)	3.7% (1)	0% (0)	0% (0)	0% (0)	0.09	3.02 [0.79–11.50]
**Death**	1.1% (7)	2.1% (6)	0.3% (1)	0.5% (1)	0% (0)	0% (0)	0% (0)	0% (0)	0% (0)	0.04	6.80 [0.81–56.77]

ICU = intensive care unit; OR = odds ratio; Bi-pap = bilevel positive airway pressure.

There was no difference between patients infected with pH1N1 or another respiratory viruses regarding admission to an intensive care unit, use of pressors or requirement for intubation (Table 5). However, individuals with pH1N1 were more likely to require bilevel positive airway pressure ventilation. Individuals with other respiratory viruses were more likely to receive inhaled bronchodilators or nebulizer therapy in the emergency department. Seven patients (1.1%) in the cohort died and these individuals were more likely to be infected with pH1N1 (2.1% vs. 0.3%, p<0.05).

In patients with cough, the presence of subjective fever/chills independently increased the likelihood of pH1N1 infection (Table 6). In patients with cough and gastrointestinal complaints, subjective fever/chills independently increased the likelihood of having pH1N1. Using fever alone did not raise the likelihood of having influenza infection versus another respiratory virus. Using age as a covariate, patients 19 to 59 years of age had the highest likelihood of presenting with pH1N1 compared to other age groups.

**Table 6:** Likelihood of patients with clinical characteristics having pandemic 2009 influenza A (pH1N1) using multiple logistic regressions.

Likelihood of having pandemic 2009 influenza A (H1N1)	OR	[95% CI]
Subjective fever/chills in patients with cough and gastrointestinal complaints	9.96	[4.04–24.59]
Subjective fever/chills in patients with cough and no gastrointestinal complaints	4.02	[2.03–7.99]
Subjective fever/chills in patients without cough or gastrointestinal complaints	0.70	[0.20–2.47]
Cough in patients with subjective fevers/chills	1.79	[0.97–3.31]
Cough in patients without subjective fevers/chills	0.31	[0.09–1.01]
Gastrointestinal complaints in patients with subjective fevers/chills	2.29	[1.57–3.34]
Gastrointestinal complaints in patients without subjective fevers/chills	0.92	[0.34–2.54]
≥60 years-old versus 19–59 years-old	0.22	[0.11–0.44]
≥60 years-old versus 5–18 years-old	0.27	[0.13–0.56]
≥60 years-old versus <5 years-old	1.30	[0.65–2.58]
19–59 years-old versus 5–18 years-old	1.26	[0.78–2.04]
19–59 years-old versus <5 years-old	5.98	[3.86–9.24]
5–18 years-old versus <5 years-old	4.75	[3.02–7.47]

OR = odds ratio; CI = confidence intervals.

An age-adjusted analysis was performed to assess if any factors were found that significantly impacted the likelihood of patients presenting with pH1N1. Age was a significant variable for those patients who had cancer (<5 years OR 5.7, 95% CI 0.51–63.6; 5–18 years OR 0.26, 95% CI 0.023–3.0; 19 and older OR 0.11, 95% CI 0.041–0.31), neurological symptoms (<5 years OR 2.67, 95% CI 0.98–7.3; 5–18 years OR 1.27, 95% CI 0.51–3.17; 19 and older OR 0.52 95% CI 0.23–1.17), or dyspnea (<5 years OR 0.33, 95% CI 0.18–0.61; 5–18 years OR 0.26, 95% CI 0.29–1.21; 19 and older OR 0.95, 95% CI 0.56–1.60). Age was also found to have a significant affect on sodium, creatinine, hematocrit, heart rate, diastolic blood pressure, the use of nebulizers, and the administration of antibiotics across different age groups (<5 years, 5–18 years, and 19 years and older). Patients with pH1N1 who were younger tended to be given more antibiotics (<5 years OR 1.30, 95% CI 0.73–2.33; 5–18 years OR 1.63, 95% CI 0.82–3.2; 19 and older OR 0.42, 95% CI 0.24–0.74). Age did not have a significant impact on any other variables.

## Discussion

In patients with viral respiratory infections, diagnosis of influenza is important to provide timely and efficient treatment with neuraminidase inhibitors. Rapid antigen tests were insensitive in the diagnosis of influenza during the 2009–2010 pandemic season [29], [6], [30]. Furthermore, these tests could not distinguish between different influenza A subtypes [31]. Seasonal influenza A (H1N1) was resistant to oseltamivir, whereas pH1N1 was not, making this a critical distinction [32]. While state public health labs had a CDC-based PCR assay for distinguishing influenza subtypes, an FDA-cleared product for clinical laboratories was delayed [33]. Therefore, many institutions, including our own, implemented a molecular-based test to diagnose influenza A [34]. The Luminex xTAG RVP was highly sensitive and able to distinguish 18 viruses causing respiratory infections, including different influenza subtypes.

With the introduction and effectiveness of molecular testing, one goal is more efficient use of antimicrobials and the reduction of unnecessary antibiotic use. Despite the relatively rapid turnaround time of the PCR-based tests, greater than half of the patients in our cohort with documented viral infections received antibacterial agents, presumably for empiric coverage of bacterial pneumonia. Furthermore, almost half of patients without influenza received oseltamivir. As such, implementation of rapid diagnostic testing for respiratory pathogens alone may not limit antibiotic use without other interventions. These data suggest overuse of antibacterial and antiviral agents and an opportunity for a robust antimicrobial stewardship program.

Clinical characteristics of hospitalized patients with pH1N1 were variable. Fever and cough, two criteria for ILI, often occur in influenza A patients [3], [14], [12], [15]–[18], [20], [21], [24]. Although more patients with pH1N1 presented with fever and sore throat compared to those with other viruses in our population, the difference was not enough to make a firm clinical diagnosis of influenza. Furthermore, there was no significant difference in cough alone between patients infected with pH1N1 and other respiratory viruses. However, fever, cough, and gastrointestinal symptoms increased the likelihood of pH1N1 almost 10-fold in the pediatric population and may be useful as a preliminary guide to prompt clinicians to treat influenza infection. Chest radiographs may be useful in diagnosing superimposed bacterial infection. While airspace disease was observed more often in patients with non-influenza viruses, there were no chest radiographic findings that distinguish influenza infection. Over half of patients with pH1N1 had non-specific findings on chest radiograph as previously reported [24].

Our study supports previous findings that pH1N1 tends to infect younger adults, sparing the elderly and young children [3], [14], [15], [17], [19], [18], [20], [21]. We found lower rates of influenza from nursing home patients reflecting this age distribution. Of those that died or were hospitalized, many had co-morbidities as previously reported [15], [17], [19], [21], [35], [36]. In contrast to other studies [15], [37], [38], [21], [19], we did not find a high infection or mortality rate during pregnancy but our study was underpowered due to the low number of pregnant women in our cohort.

The mortality rate of 2.1% for hospitalized patients with pH1N1 infection in our cohort was lower than other reports [14], [15]. Despite this, it was significantly higher than the mortality associated with other respiratory viral infections and highlights the importance of accurate diagnosis and early treatment of influenza infection.

Aside from the retrospective nature of our study, a potential limitation was the small number of pregnant women likely due to the presence of a neighboring obstetrics and gynecologic hospital. A second limitation was the time period for which patients presenting with ILI were evaluated (6 weeks at the peak of the pandemic) whereas a typical respiratory season would be for several months and include a greater variety of viruses, especially in the pediatric population. In fact, our data (not shown) does indicate that after the pandemic wave at our institution, a typical peak for RSV, metapneumovirus and parainfluenza viruses followed the presence of pH1N1, much like the rest of the country. A third limitation was that pH1N1 confirmatory testing was not performed for all non-subtypeable influenza A viruses. However, recent literature suggests that 100% of non-subtypeable influenza A H1 identified by the xTAG RVP was pH1N1 [39] and that misinterpretation is uncommon [40]. In addition, our initial investigation of a large number of strains early in the pandemic with the CDC PCR assay confirmed these findings. Many prior studies only assessed the clinical characteristics of patients with influenza or compared to individuals who's respiratory tests were negative for influenza, but they did not further delineate those without influenza or positive for another virus [14], [16], [18], [20], [21], [25], [26]. We set out to compare pandemic 2009 influenza A (H1N1) to other respiratory viruses in patients with ILI. To our knowledge, these results provide the first comparison of clinical characteristics between pH1N1 and other common respiratory viruses.

While a specific clinical presentation could not confirm pH1N1 in patients with cough and gastrointestinal complaints, the presence of subjective fever and/or chills increased the likelihood of pH1N1 infection versus another virus. Respiratory infection with pH1N1 infection more often resulted in death compared to other respiratory viruses and should be treated aggressively with supportive measures and antiviral medications. Despite the use of RVP testing, many influenza-infected patients received antibacterial agents and many patients without influenza received antivirals. Use of a highly accurate RVP in conjunction with a robust antimicrobial stewardship program will be necessary to assure prudent antibacterial and antiviral agent use in the future.

## Materials and Methods

### Ethics

The study was approved by the Rhode Island Hospital institutional review board. A waiver of informed consent was obtained before onset of the study.

### Study Design

A retrospective review was performed of all individuals presenting to our hospital system between October 16, 2009 and December 1, 2009 who had a positive respiratory viral panel (RVP, Luminex xTAG®; Luminex Corporation, Austin, TX) result from a nasopharyngeal swab specimen and who were subsequently hospitalized. Our hospital system consists of Rhode Island Hospital, a tertiary care center licensed for 719 beds, including Hasbro Children's Hospital, as well as The Miriam, Newport and Bradley Hospitals licensed for 247, 129 and 60 beds, respectively. All respiratory specimens were processed in the microbiology facility at Rhode Island Hospital. Our 18-virus panel detected influenza A/B (H1, H3, and non-subtypeable A consistent with pH1N1), respiratory syncytial virus A and B, adenovirus, metapneumovirus, rhinovirus/enterovirus, parainfluenza 1,2,3,4 and coronaviruses (NL63, OC43, HKU1, and 229E). The panel determined influenza A as seasonal human influenza A (H1N1), seasonal human influenza A (H3N2) or a non-subtypeable influenza A virus consistent with pH1N1. The Rhode Island Department of Health (DOH) confirmed the initial 30 specimens detected by the xTAG RVP as non-subtypeable influenza A H1 as pH1N1, utilizing primers and probes distributed by the Center for Disease Control and Prevention (CDC). Thus, subsequent non-subtypeable influenza A H1 detected by the RVP were reported as pH1N1.

### Statistical Analysis

Medical records of all cases were reviewed. Initial chest radiographs and subsequent chest CTs were reviewed and interpreted independently by three board-certified radiologists. Consensuses on all findings were reached. Logistic regressions were used to examine the relationships between variables and patients testing positive for pH1N1 compared with patients testing positive for a different respiratory virus. Subsequently, a series of multiple logistic regressions were constructed based on integrating the results from previous literature and our logistic regression results. Individual interactions between variables were checked and those with p>0.15 were retained, arriving at a final model. Special effort was placed on using symptoms and other clinical information. Co-linearity between predictors was minimized by forming theoretically and clinically guided composites as needed.

All predictors were tested for an interaction with different age categories (<5 years, 5–18 years, 19 years and older) with regards to predicting pH1N1 in logistic regressions. Models included main effects for the predictor, age, and the interaction of the two. When a statistically significant interaction was detected, the simple effects of the predictors were described in terms of their effects within age categories. Those which did not significantly interact with age were described in terms of their main effect.
